# Biological correlates of cancer-related fatigue in older male cancer survivors

**DOI:** 10.1038/s41398-026-04274-1

**Published:** 2026-07-17

**Authors:** Samuel Tundealao, Michael R. Irwin, Steve Cole, Cindy K. Blair, Shou-En Lu, Biren Saraiya, Isaac Y. Kim, Dolores D. Guest, Yong Lin, Jinghua An, Evelyn Arana-Chicas, Chunxia Chen, Elizabeth Handorf, Antoinette Stroup, Yibin Kang, Wadih Arap, Anita Y. Kinney

**Affiliations:** 1 Department of Biostatistics and Epidemiology, Rutgers University School of Public Health, Piscataway, NJ USA; 2 Rutgers Cancer Institute, New Brunswick, NJ USA; 3 Department of Psychiatry, David Geffen School of Medicine, University of California, Los Angeles, CA USA; 4 Cousins Center for Psychoneuroimmunology, Semel Institute for Neurosciences, University of California-Los Angeles, Los Angeles, CA USA; 5 University of New Mexico Comprehensive Cancer Center, Albuquerque, NM USA; 6 Department of Internal Medicine, University of New Mexico, Albuquerque, NM USA; 7 Division of Medical Oncology, Section of Solid Tumors, Robert Wood Johnson Medical School, Rutgers University, New Brunswick, NJ USA; 8 Yale New Haven Hospital, New Haven, CT USA; 9 Indiana University School of Nursing, Indianapolis, IN USA; 10 Division of Medical Oncology, Section of Behavioral Science, Robert Wood Johnson Medical School, Rutgers University, New Brunswick, NJ USA; 11 Department of Molecular Biology and Ludwig Institute for Cancer Research-Princeton Branch, Princeton University, Princeton, NJ USA; 12 Rutgers Cancer Institute, Newark, NJ USA; 13 Division of Hematology and Oncology, Department of Medicine, Rutgers New Jersey Medical School, Newark, NJ USA

**Keywords:** Physiology, Biomarkers, Genetics

## Abstract

Older male cancer survivors often experience chronic fatigue. Although the pathogenesis of cancer-related fatigue (CRF) remains unclear, circulating inflammatory markers and gene expression profiles may provide insights into the underlying biological mechanisms of persistent CRF. We examined the potential biological correlates of CRF in older male cancer survivors. This is a secondary analysis of baseline data from a randomized controlled trial that examined the effects of Tai Chi Qigong on fatigue and inflammation biology among 107 older male cancer survivors (≥ 55 years). Fatigue was assessed with the Functional Assessment of Chronic Illness-Fatigue Scale. Blood samples were collected for circulating inflammatory biomarkers (C-reactive protein, IL-6, IL-8, IL-10, IFN-γ, TNF-α), and for genome-wide transcriptional profiling. Pearson’s correlation and multivariable linear regression were used to assess these relationships while adjusting for sociodemographic and clinical factors. Due to multiple testing, false discovery rate-corrected p-values were reported. Worse fatigue was significantly associated with higher levels of CRP (p = 0.011), IL-6 (p = 0.002), and TNF-α (p = 0.010). Fatigue was also significantly correlated with increased expression of three immune-related genes, namely [CXCR4 (p = 0.023), IRF-7 (p = 0.009), and TGM2 (p = 0.018)], one mitochondrial-related gene [HSPA2 (p = 0.001)], one transcription factor gene [ETS1 (p = 0.037)], one neuropeptide [OPRL1 (0.036)], and decreased expression of two immune-related genes [LY6E (p = 0.029), COMMD9 (p = 0.034)], and three RNA/DNA processing genes [SNORD89 (p < 0.001), TFIP11 (p = 0.017), and UNG (p = 0.003)]. CRF was associated with inflammatory profiles and coordinated transcriptional shifts involving immune activation, neuro-immune signaling, mitochondrial dysfunction, and impaired nucleic acid processing, thereby offering a coherent biological context for CRF in this population. **Clinical Trial Registration**: The HERO Trial was registered in the NIH Clinical Trials Registry on November 17, 2017 (NCT03345563).

## Introduction

Approximately 90% of male cancer survivors are 55 years old or older [1]. Many have challenges coping with short-term and long-term cancer and cancer treatment-related effects [2], which are often compounded with physiological declines and comorbidities associated with aging [3]. Cancer-related fatigue (CRF) is one of the most common symptoms experienced by survivors throughout the cancer care continuum [4]. Furthermore, CRF is the least understood symptom by both patients and healthcare providers [5]. The National Comprehensive Cancer Network has defined CRF as “a distressing, persistent, subjective sense of physical, emotional, and/or cognitive tiredness or exhaustion related to cancer or cancer treatment that is not proportional to recent activity and interferes with usual functioning” [5]. Unlike fatigue experienced by healthy individuals, CRF is not relieved by rest or sleep and can last for months or even years after therapy has been completed [5]. CRF is highly prevalent, affecting up to 90% of all cancer patients at diagnosis and during treatment, as well as up to 50% of survivors months or years after the completion of cancer treatment therapy [6]. Cancer and its treatment—such as surgery, radiation therapy, chemotherapy, immunotherapy, and hormonal therapy—can trigger systemic physiological alterations such as inflammation, mitochondrial dysfunction, and hormonal disruptions, which have been implicated in CRF [7]. These biological perturbations highlight the intricate features of CRF and underscore the need to further understand the underlying mechanisms.

Despite a substantial body of research, the etiology of CRF still remains unclear. Early studies ascribed CRF to emotional distress and/or deconditioning associated with cancer and its treatment [8]. However, biological mechanisms may also contribute to persistent CRF. Indeed, an accumulation of evidence has indicated that biological dysregulation, especially involving inflammatory and neuroendocrine systems, plays a role in the etiology of CRF. Many studies have shown that higher levels of circulating inflammatory cytokines and acute-phase proteins are associated with more severe fatigue in cancer patients [9–13]. Specifically, the pathophysiology of CRF has been consistently associated with higher levels of Interleukin (IL)-1β, IL-6, IL-8, IL-10, C-reactive protein (CRP), and tumor necrosis factor-alpha (TNF-α) [14–16]. Elevated serum levels of these cytokines indicate that activation of innate immune signaling pathways might be associated with CRF [17, 18]. Furthermore, chemokines such as C-X-C motif chemokine (CXCL)-9, CXCL10, and C-C motif chemokine (CCL2) have also been implicated in leucocyte trafficking and chronic low-grade inflammation [19–21], thereby reinforcing the concept that CRF may be a systemic manifestation of a chronic immune modulation. However, there have been inconsistencies noted across studies regarding the types of circulating inflammatory markers associated with CRF, likely due to the interaction of multiple biological pathways in a network rather than changes in a single immune or molecular factor.

Alongside inflammation, advances in genomic and transcriptomic profiling have identified numerous genes and signaling pathways that may be implicated in CRF. Genetic polymorphisms and differential expression in genes associated with cytokine signaling (e.g., IL-6, IL-8, TNF-α, IL-1B), stress response (e.g., NR3C1, COMT, SIRT1), circadian rhythm (e.g., CLOCK, PER1, PER2), and mitochondrial regulation (e.g., P2RY1, ATP5E, NDUFS5) have been correlated with the severity of fatigue among cancer survivors [22–26]. In addition, changes in the transcription of interferon-related factors (e.g., IRF1) and nuclear receptors (e.g., NR4A1) have provided further evidence of immune–metabolic interactions in the manifestation of CRF [27, 28]. These findings collectively support a multifactorial system in which inflammatory, metabolic, and neuroendocrine pathways converge to induce CRF. Nonetheless, one should note that the preponderance of CRF research has been undertaken among female cancer survivors, especially those with breast cancer, or within mixed-gender cohorts. Research in men is far more limited, and the biological mechanisms of CRF in men, who are biologically, hormonally, and therapeutically distinct from women, are relatively unknown.

The identification of gene expression clusters or co-expressed molecular networks associated with CRF is crucial for more effectively elucidating its multifaceted biology. Functional cluster-based gene expression analysis can identify intercorrelated gene sets that may synergistically influence immunological, metabolic, transcriptional, circadian, and other pathways potentially associated with CRF. Light et al. [29] conducted a landmark study among prostate cancer patients and identified two functional gene clusters based on mRNA intercorrelations: Cluster I, associated with energy and adrenergic pathways, and Cluster II, associated with inflammation and vasodilation/metabolite sensing [29]. Their findings further strengthen the notion that CRF is not driven by a singular molecular cause, but rather by the co-occurring dysregulation of interconnected pathways.

Building on the existing literature, this study examined potential biological correlates of CRF in older male cancer survivors. Specifically, we investigated the correlation between circulating inflammatory biomarkers, gene expression profiles, and the clinical magnitude of CRF. The key goal of this study was to test the hypothesis that older male cancer survivors with worse fatigue will have higher systemic inflammation and unfavorable expression of fatigue-associated genes (depending on the gene’s pathway) compared to those with lower fatigue levels. Genes from five pathways described by Light et al. [29] were examined. These include (1) adrenergic, monoamine, and peptides; (2) immune response and inflammation; (3) sensory ion channel receptors responding to adenosine triphosphate (ATP) and other metabolites of muscle activity; (4) mitochondrial and other genes involved in lipid/energy metabolism; and (5) transcription and growth factors. In contrast to whole-genome microarray studies, which utilize thousands of mRNA genes, many of which lack prior justification for a potential association with CRF, our study employed a targeted selection of smaller gene sets that comprise pathways demonstrated to modulate CRF and fatigue generally. We also identified functional gene clusters associated with CRF based on mRNA intercorrelations among older male cancer survivors.

## Subjects and methods

### Study design and participants

This study used baseline data from the Health Empowerment and Recovery Outcomes (HERO) trial, a 3-arm single-blind randomized controlled trial that examined the effects of Tai Chi Qigong, exercise-intensity-matched, and usual care on fatigue, inflammation biology, and gene expression profiles in older male cancer survivors [30, 31]. The trial recruited male cancer survivors (n = 113) from the Rutgers Cancer Institute in New Jersey and the University of New Mexico Comprehensive Cancer Center in New Mexico, between June 2017 and January 2022. This cross-sectional study was a secondary analysis of HERO baseline biomarker data.

The inclusion criteria for the HERO trial included (i) age 55 years and older; (ii) a personal history of pathologically confirmed local or regional stage cancer of one or more of the following sites: prostate (also included either biochemical recurrence or metastatic disease), colorectal, oropharyngeal, lung, small intestine, thyroid, bladder, renal pelvis and kidney; (iii) have completed cancer therapy for more than three months (except oral hormonal manipulation therapy); (iv) if on androgen-deprivation therapy (ADT) or other hormone therapy for prostate cancer, must have been taking it for over four months; (v) Patients have at least a 1-year life expectancy; (vi) fatigued, i.e., had a score of ≥ 9 (T-score M = 51, SE = 2.5, SD = 10) on the Patient-Reported Outcomes Measurement Information System Fatigue 4a scale [32] or ≤ 13 on the Medical Health Outcome Study Short Form Vitality/Fatigue subscale [33]; (vii) engaged in less than 150 min of vigorous exercise per week within the last three months; (viii) able to travel to on-site classes and assessments (pre-COVID-19 pandemic only); and (ix) can read and speak the English language.

Participants were excluded if they (i) were being treated for hematological malignancies because of the potential for confounding the study’s biological measures; (ii) scored > 12 or indicated suicidality on the Patient Health Questionnaire PHQ-9 scale [34]; (iii) scored ≤ 50 in the Karnofsky Performance Status (KPS) Scale [35]; (iv) reported engaging in regular practice of body-mind therapy or other complementary therapies two-or-three-times a week for two months over the past one year; or (v) had cancer that has metastasized to lungs, brain, or liver.

### Sample collection and processing

Fasting blood samples were collected from the participants at the baseline assessment. Among the older male cancer survivor enrollees (n = 113), six had phlebotomy failure or poor-quality sample at baseline, resulting in a revised cohort sample size (n = 107). Participants were instructed to abstain from exercise and certain medications [such as corticosteroids and those that can affect the sympathetic nervous system, hypothalamic-pituitary-adrenal axis (HPA), or cytokine activity] for 72 h (with their physician’s consent) before the blood draws [36]. These are standard guidelines for inflammation-related and adrenergic gene expression samples. A certified phlebotomist collected blood samples at in-person phlebotomy visits. Specimens were processed and stored at −80 °C, then dispatched to the designated laboratories for further processing.

### Measures

#### Fatigue

Fatigue was evaluated using the Functional Assessment of Chronic Illness-Fatigue Scale (FACIT-F). The scale consists of thirteen items that assess fatigue levels during usual activities over the past seven days. FACIT-F score ranges from 0 to 52, with lower scores indicating more fatigue (Cronbach’s alpha = 0.87) [37].

#### Circulatory inflammatory markers

Frozen plasma samples were thawed and analyzed by high-sensitivity enzyme-linked immunosorbent assay (ELISA) for CRP (Immundiagnostik, ALPCO Immunoassays), and by a custom high-sensitivity cytokine multiplex assay, which included IL-6, IL-8, IL-10, IFN-γ, and TNF-α (Luminex Performance Assays, R&D Systems). Quality control was monitored in all assays by using internal laboratory control specimens. Systemic inflammation was assessed with CRP (in mg/L), IL-6, IL-8, IL-10, IFN-γ, and TNF-α (all in pg/mL).

#### Gene expression profiles

RNA was extracted by using Qiagen RNeasy, reverse-transcribed into cDNA with a high-efficiency mRNA-targeted enzyme system (Lexogen QuantSeq 3’ FWD) and subsequently sequenced on an Illumina NextSeq equipment (Illumina HiSeq4000 system). The sequencing targeted 5 million reads per sample, with each read mapped to the GRCh38 human reference transcriptome utilizing the STAR aligner. The average achieved mean read per sample and mapping rate at baseline were 8.9 million and 84.3%, respectively. Quality control was conducted to identify samples with poor mapping rates (<70%), sequencing reads (<5 million), and signal-to-noise ratios (r < 0.90). The baseline flag rate was 8.4%, yielding 98 high-quality sample results available for gene expression correlation analysis. This study analyzed 160 CRF-relevant genes, which were identified from the previous literature [10, 12, 22–29, 38–50]. These genes were categorized into five important general pathways as described by Light et al and presented earlier [29]. The pathways, genes, and hypothesized direction of correlation with CRF were presented in Supplementary Table 1.

#### Other covariates

Information on sociodemographic (age, race, ethnicity, marital status, education level, annual household income, and health insurance) and clinical (cancer diagnosis, years since diagnosis, stage of disease, type of cancer treatment, and hormone therapy such as ADT) factors was collected via self-report in the baseline survey and/or the patients’ electronic health medical records. Body mass index (BMI) in kg/m^2^ was estimated from height (m) and weight (kg) measurements obtained during the participant’s baseline in-person assessment.

### Data analysis

Descriptive statistics were used to characterize participants’ sociodemographic and clinical factors (mean and standard deviation (SD) for continuous variables, and count and percentage for categorical variables). Inflammatory markers (CRP, IL-6, IL-8, IL-10, IFN-γ, and TNF-α) exhibited a non-normal distribution (right-skewed) and were therefore log-transformed. Gene transcripts were floored at one transcript-per-million to minimize spurious low-range variability, log_2_-transformed to stabilize level-dependent variance within genes, and z-score-transformed to stabilize variance across genes.

Pearson’s correlation coefficient (r) was used to assess the association between circulating inflammatory markers, fatigue-related genes, and CRF (measured with FACIT-F score, a continuous variable) among the men at the baseline assessment. Higher FACIT-F scores signify less fatigue, while lower scores indicate increased fatigue; hence, a positive correlation (r > 0) indicates a negative association, while a negative correlation (r < 0) suggests a positive association between the biomarkers and CRF. The 95% confidence intervals (CI) for r were calculated by using Fisher’s Z transformation. Multivariable linear regression was then performed to examine the association between CRF and each inflammatory marker and fatigue-related gene while adjusting for socio-demographic (age, race, ethnicity, marital status, education, household income, health insurance status) and clinical factors (cancer type, years since diagnosis, stage of disease, hormone therapy use). Multiple comparisons were controlled by using the false-discovery rate (FDR) [51]. Effect sizes (r and regression coefficient *β*) and 95% CIs were reported for systemic inflammation biomarkers and genes significantly associated with fatigue. Only FDR-corrected p-values were reported. These analyses were conducted using SAS version 9.4.

To explore functional gene expression clusters associated with CRF via mRNA intercorrelation, we first computed an intercorrelation matrix by calculating pairwise Pearson correlations across all 160 gene variables. We then assessed statistical significance and filtered the results to retain only significant correlations (p < 0.05). The resulting correlation matrix was converted to an edge list in a long format suitable for network analysis. By using these significant pairwise relationships, we built and visualized a correlation network in which nodes represented genes and edges reflected significant intercorrelations. Functional clusters within this network were subsequently identified through multi-level optimization of modularity to detect community structure [52], and nodes corresponding to each cluster were extracted for further biological interpretation. mRNA intercorrelation analyses were performed by using “corrplot”, “igraph”, and “ggraph” packages on R Studio version 4.5.2.

## Results

### Participants characteristics

Socio-demographic and clinical factors of the participants are summarized in Table 1. The mean age of the older male cancer survivors was 69.4 years (SD = 6.99). Most participants were non-Hispanic (88.8%), White (78.5%), married or in a domestic partnership (77.4%), had health insurance (97.2%), and had an annual household income of $70,000 or more (63.2%). The average BMI was 30.2 (SD = 6.61). Eighty-eight percent (n = 95) had prostate cancer. Participants were, on average, 4 years post-diagnosis (median = 4; IQR = 6). Twenty-nine (27.1%) had distant or biochemical disease, and 28 (26.2%) were on ADT or other hormone therapy.

**Table 1:** Sociodemographic and clinical features of participants.

Characteristics	N (%)
	107 (100)
**Age** (Mean, SD) years	69.4 (6.99)
**Hispanic Ethnicity**	
No	95 (88.8)
Yes	12 (11.2)
**Race**	
White	84 (78.5)
Black/African American	12 (11.2)
Other^a^	11 (10.3)
**Marital Status**	
Single/Divorced/Separated/ Widowed	24 (22.6)
Married/Domestic Partnership	82 (77.4)
Missing	1
**Education level**	
Less than high school/High school grad/GED	15 (14.2)
Some college/Assoc. Degree/Vocational School	30 (28.3)
Bachelor’s degree or higher	61 (57.5)
Missing	1
**Annual household income**	
<$30,000	10 (11.5)
$30,000–$49,999	8 (9.2)
$50,000–$69,999	14 (16.1)
$70,000 or more	55 (63.2)
Didn’t want to report or missing	20
**Health insurance**	
No	3 (2.8)
Yes	103 (97.2)
Missing	1
**Body Mass Index** (Mean, SD)	30.2 (6.61)
**Cancer diagnosis**	
Prostate only	84 (78.5)
Prostate and other types of invasive cancer(s)	11 (10.3)
Other cancer type(s)	12 (11.2)
**Years since initial diagnosis** (Median, IQR)	4.0 (6.0)
**Stage of Disease**	
Local	69 (64.5)
Regional	9 (8.4)
Distant/Biochemical Disease	29 (27.1)
**Currently taking hormone therapy**	
No	79 (73.8)
Yes	28 (26.2)

^a^“Other” races include: 2 American Native or Alaska Native, 1 Chinese, 1 Southeast Asian, 1 Filipino, 1 multiracial, and 5 Hispanic participants did not specify their race.

### Inflammatory markers

**Fig. 1:**
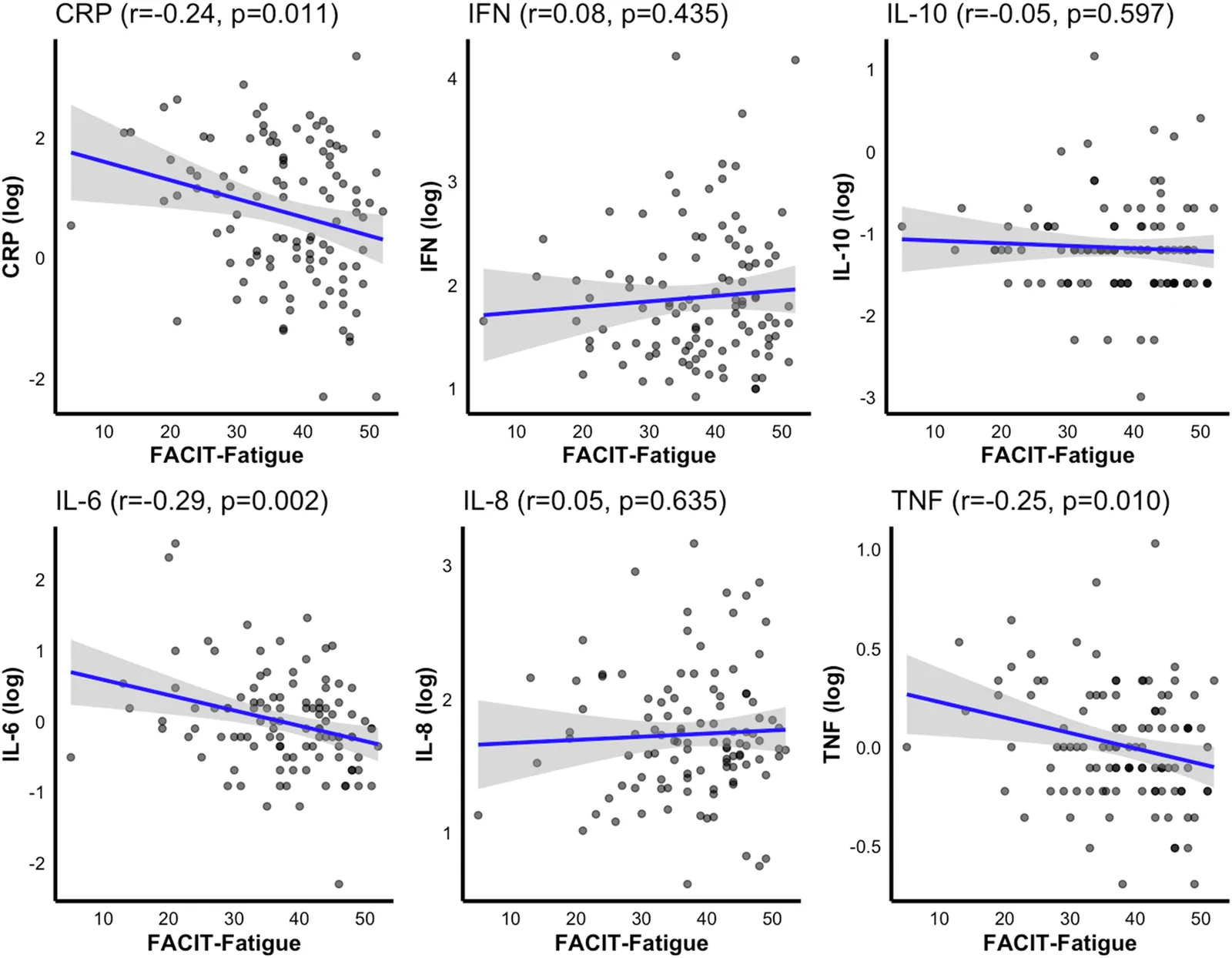
Plots showing correlation between CRF and circulating inflammatory markers.

Worse fatigue was significantly associated with higher levels of CRP (r = −0.24; CI: −0.41–−0.06; p = 0.011), IL-6 (r = −0.29; CI: −0.46–−0.11; p = 0.002), and TNF-α (r = −0.25; CI: −0.42–−0.06; p = 0.010) (Fig. 1, Supplementary Table 2). The adjusted analyses yielded similar effects (Supplementary Table 2). No association was found between IL-8, IL-10, IFN-γ, and CRF.

### Gene expression profiles

#### Adrenergic, monoamine, and peptides

Higher fatigue was also associated with higher expression of neuropeptide [OPRL1 (r = 0.21; CI: 0.01–0.38; p = 0.036)] (Table 2).

**Table 2:** Genes showing significant correlation with fatigue severity.

Genes	Pathway	Bivariate		Multivariable^a^	
		r (95% CI)^b^	*p*-value	β (95% CI)	*p*-value
**Decreased mRNA expression**					
COMMD9	Immune response and inflammation	0.21 (0.02–0.39)	0.034	0.03 (0.001–0.05)	0.048
LY6E	Immune response and inflammation	0.22 (0.02–0.39)	0.029	0.01 (−0.03–0.04)	0.808
SNORD89	Transcription and growth factors	0.37 (0.18–0.52)	<0.001	0.91 (0.15–1.67)	0.015
TFIP11	Transcription and growth factors	0.24 (0.04–0.41)	0.017	0.01 (−0.025–0.04)	0.593
UNG	Transcription and growth factors	0.29 (0.10–0.46)	0.003	0.04 (0.01–0.07)	0.029
**Increased mRNA expression**					
HSPA2	Mitochondria and lipid/energy metabolism	−0.32 (−0.49–−0.13)	0.001	−0.003 (−0.01–0.01)	0.484
CXCR4	Immune response and inflammation	−0.22 (−0.20–−0.03)	0.023	−0.01 (−0.03–0.02)	0.602
IRF7	Immune response and inflammation	−0.26 (−0.44–−0.07)	0.009	−0.02 (−0.04–−0.001)	0.041
ETS1	Transcription and growth factors	−0.21 (−0.38–−0.01)	0.037	−0.002 (−0.02–0.01)	0.851
OPRL1	Adrenergic, monoamines, peptides	−0.21 (−0.38–−0.01)	0.036	0.01 (−0.02–0.05)	0.546
TGM2	Immune response and inflammation	−0.23 (−0.41–−0.04)	0.018	−0.03 (−0.05–−0.003)	0.034

^a^adjusted for age, race, ethnicity, marital status, education, income, health insurance, BMI, cancer type, years since diagnosis, stage of disease, and use of hormone therapy.

^b^95% CI calculated using z Fisher’s transformation.

#### Immune response and inflammation

Higher fatigue among the older male cancer survivors was correlated with lower expression of two immune-related genes [COMMD9 (r = 0.21; CI: 0.02–0.39; p = 0.034), and LY6E (r = 0.22; CI: 0.02–0.39; p = 0.029)] and higher expression of three immune-related genes [CXCR4 (r = −0.22; CI: −0.20–−0.03; p = 0.023)] (Table 2).

#### Sensory ion channel receptors and metabolite-sensing

No gene in this pathway was correlated with fatigue among the older male cancer survivors.

#### Mitochondrial and lipid/energy metabolism

Higher fatigue was also associated with higher expression of one mitochondrial-related gene [HSPA2 (r = −0.32, CI: −0.49–−0.13; p = 0.001)] (Table 2).

#### Transcription and growth factors

Higher fatigue was correlated with lower expression of three RNA/DNA processing genes [SNORD89 (r = 0.37; CI: 0.18–0.52, p < 0.001), TFIP11 (r = 0.24; CI: 0.04–0.41; p = 0.017), and UNG (r = 0.29; CI: 0.10–0.46; p = 0.003)] and higher expression of one transcription factor gene [ETS1 (r = 0.21; CI: 0.01–0.38; p = 0.037)] (Table 2).

After adjusting for participants’ characteristics, worse fatigue was also associated with decreased expression of COMMD9, SNORD89, and UNG, and increased expression of IRF7 and TGM2.

### Functional clusters associated with CRF

**Fig. 2:**
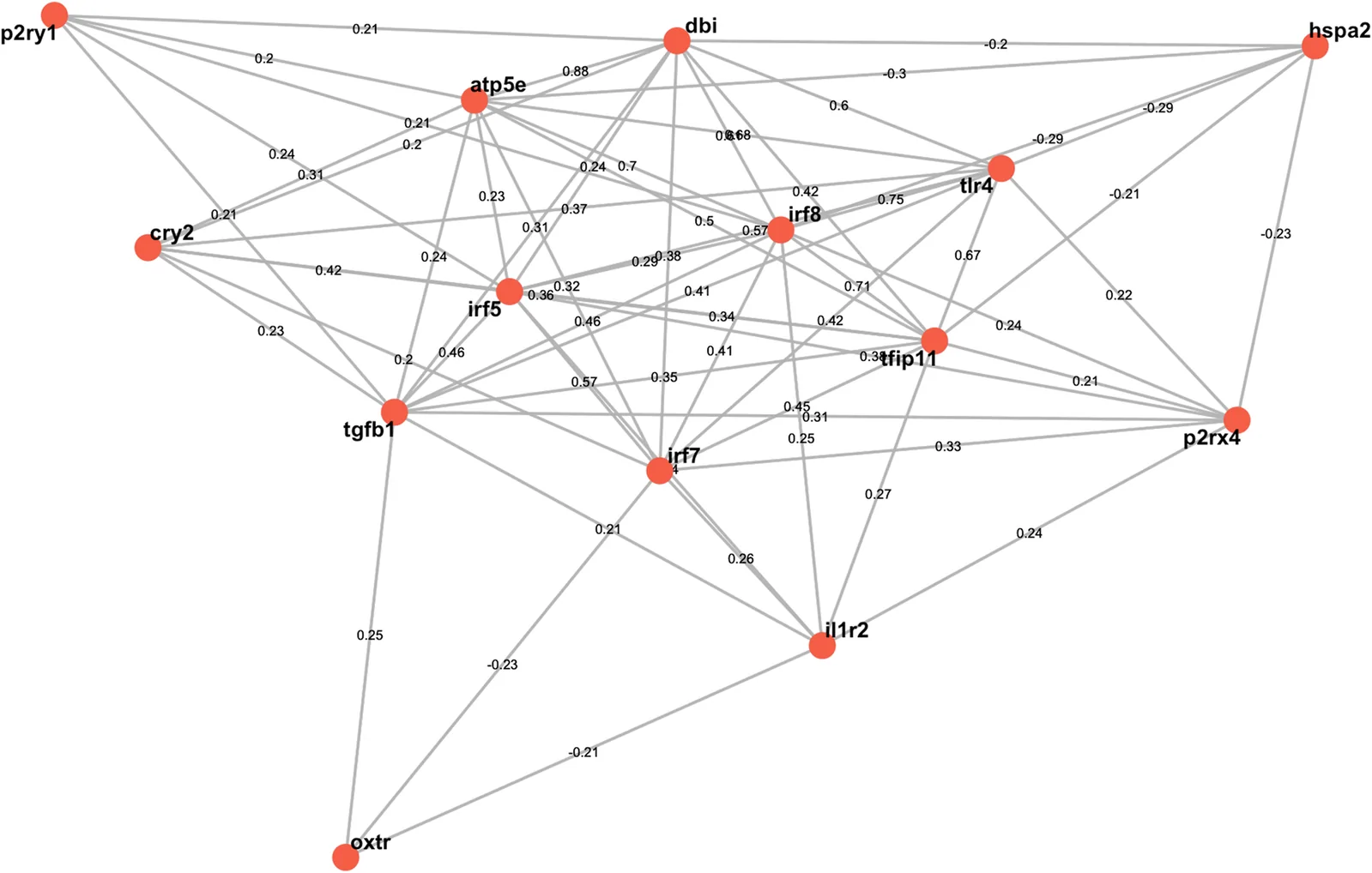
Cluster 1: Metabolic–Stress Signaling and Innate Gateways.

**Fig. 3:**
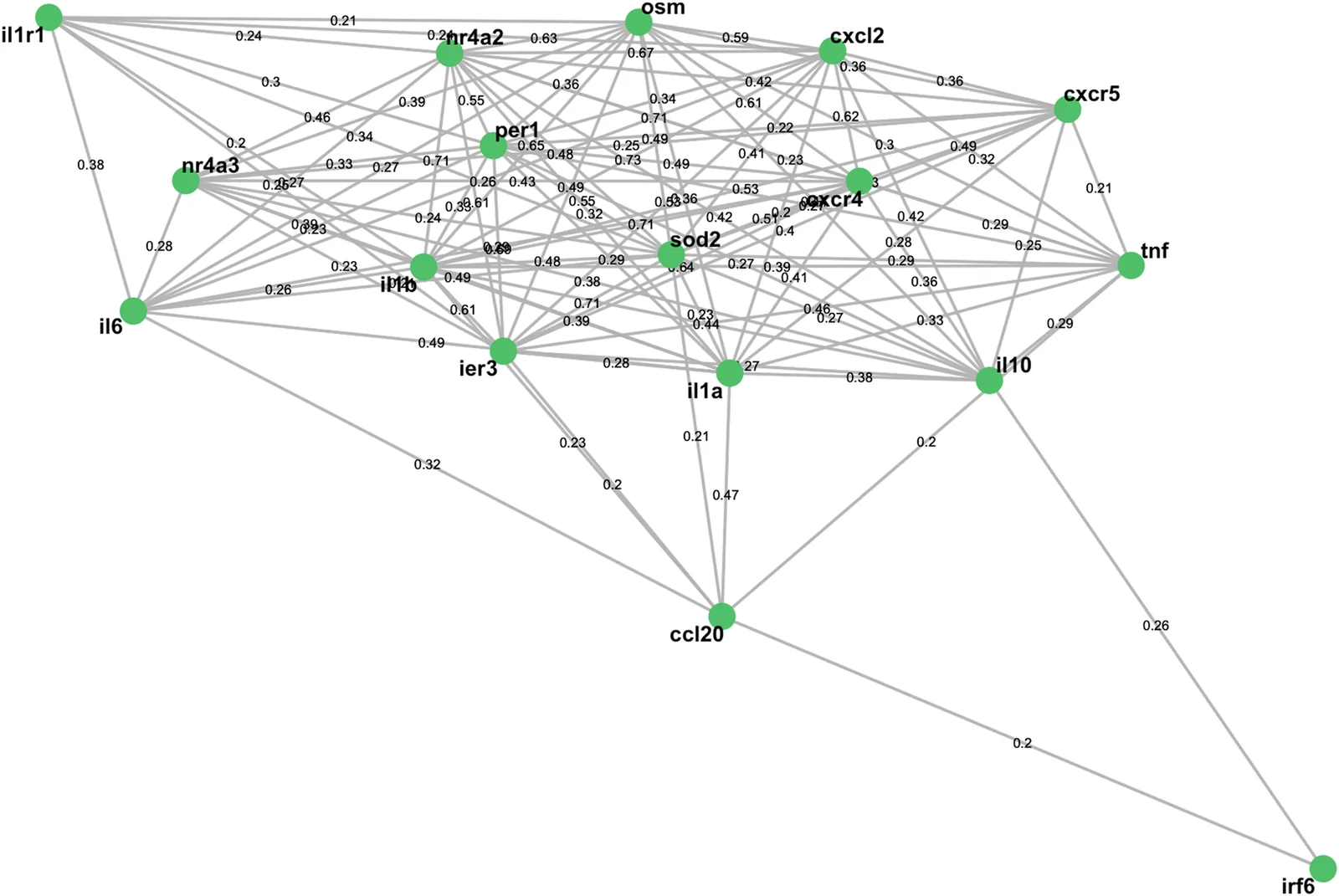
Cluster 2: Inflammatory–Neuroimmune Activation Network.

**Fig. 4:**
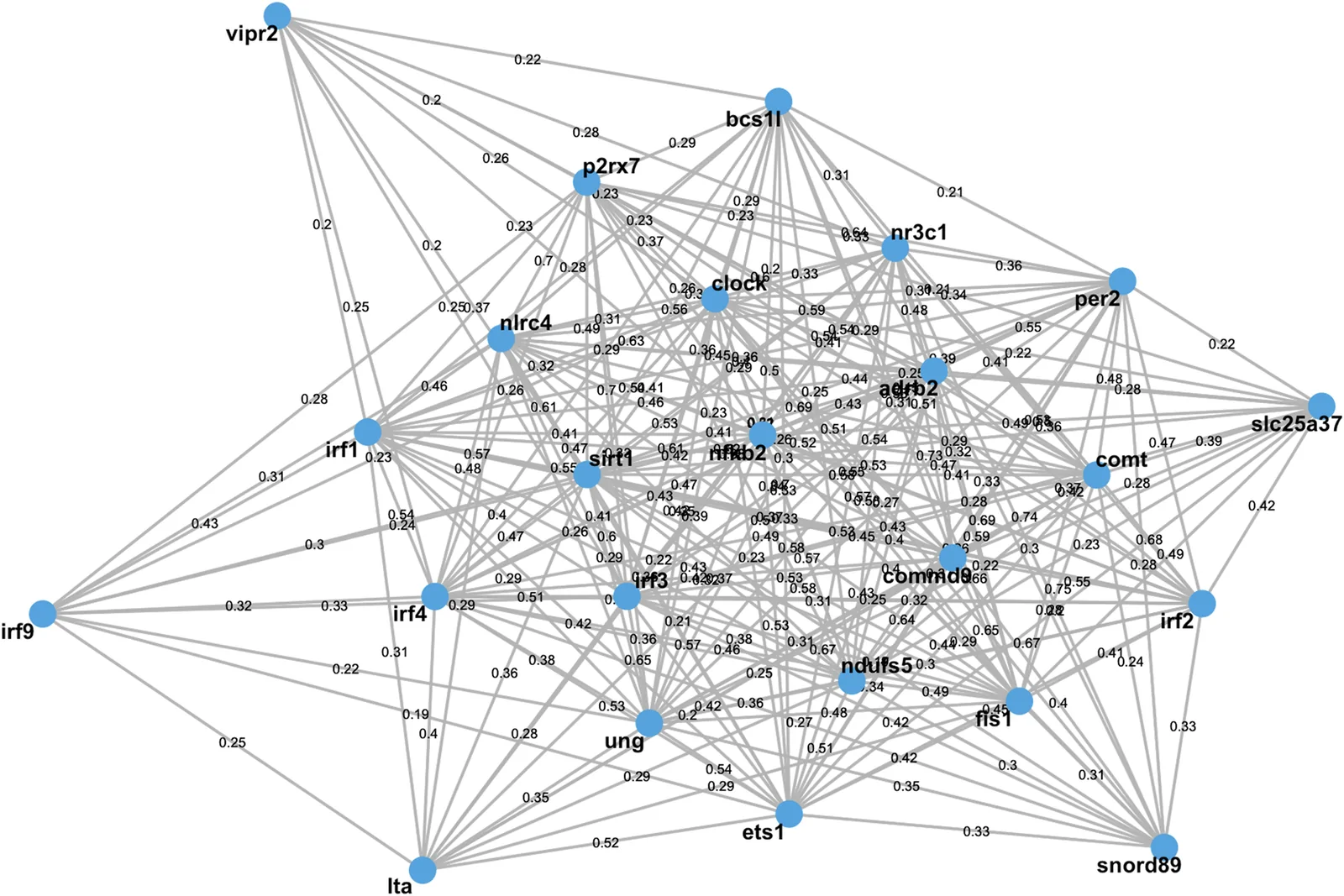
Cluster 3: Chrono-Metabolic Defense and Adaptive Remodeling Axis.

The intercorrelation of mRNA levels yielded three functional gene clusters associated with CRF (Supplementary Fig. 1). We termed cluster 1 the “*metabolic–stress signaling and innate gateways”* cluster (Fig. 2). There were fourteen genes in this cluster: P2RY1, CRY2, TGFB1, OXTR, IRF5, IRF7, IRF8, ATP5E, DBI, ILR2, TFIP11, TLR4, HSPA2, P2RX4 (r = + 0.20 to + 0.88 and −0.20 to −0.30). This cluster included two genes that were significantly expressed in association with higher fatigue (IRF7 and TFIP11). We termed cluster 2 the “*inflammatory–neuroimmune activation network”* cluster (Fig. 3). There were 16 genes: in this cluster: ILR1, IL6, NR4A3, NR4A2, IL1B, NR4A2, PER1, CCL20, SOD2, OSM, CXCL2, CXCR4, IL10, CXCR5, TNF, IRF6 (r = + 0.20 to + 0.73). This cluster included one gene that was significantly expressed in association with higher fatigue (CXCR4). We termed cluster 3 the *“chrono-metabolic defense and adaptive remodeling axis”* cluster (Fig. 4). There were twenty-four genes in this cluster: VIPR2, IRF9, LTA, IRF4, IRF1, NLRC4, P2RX7, BCS1L, CLOCK, SIRT1, IRF3, UNG, ETS1, NFKB2, ADRB2, NR3C1, COMMD9, NDUFS5, FIS1, SNORD89, COMT, PER2, IRF2, SLC25A37 (r = + 0.19 to + 0.73). This cluster included four genes that were significantly expressed in association with higher fatigue (UNG, ETS1, COMMD9, and SNORD89).

## Discussion

This study investigated the biological correlates of CRF in older male cancer survivors by analyzing related circulating inflammatory markers and gene expression profiles. Consistent with existing literature, elevated levels of CRP, IL-6, and TNF-α were significantly correlated with CRF, thereby highlighting the involvement of inflammation in the biological mechanism of CRF. Targeted gene expression analysis suggested genetic correlates of CRF across immune, neuroendocrine, transcriptional, mitochondrial, and metabolic pathways.

Independently, higher levels of CRP, TNF-α, and IL-6 were correlated with greater severity of fatigue in the male cancer survivors. CRP is an acute-phase protein produced by hepatocytes in response to IL-6, and its conjunction with neopterin and serum amyloid A (SAA) serves as a reliable sign of an active systemic inflammatory process [53]. The positive correlation between elevated CRP and CRF observed in the study corroborates findings from prior studies of survivors with various types of cancer [54–57], including those limited to male survivors [19, 23, 58], underscoring the impact of chronic low-grade inflammation on energy dysregulation and fatigue, which may even persist post-cancer treatment. This inflammation is characterized by persistent innate immune activation and diminished resilience, processes that are also exacerbated by aging and cancer treatment [59].

We identified a correlation between IL-6, a pleiotropic cytokine implicated in the acute-phase response and neuroimmune communication [60], and CRF. Our findings support evidence from prior research [61–65], suggesting a pro-inflammatory impact of IL-6 on fatigue, despite its dual role in both pro-inflammatory and anti-inflammatory functions. Studies focused exclusively on male cancer survivors have often yielded inconsistent results. For instance, Kopčalić et al. [66] identified a statistically significant positive correlation between IL-6 and CRF, while Sass et al. [19] reported an inverse correlation, while Dirksen et al. [67] found no association among prostate cancer survivors. IL-6 typically modulates sickness behavior [60] and induces central fatigue by altering hypothalamic and mesolimbic circuits, whereas also stimulating the production of CRP and other cytokines that perpetuate the low-grade inflammation associated with CRF [68]. The elevated levels of IL-6 in this older cohort of fatigued cancer survivors might also originate from the incomplete resolution of immune activation induced by cancer or its treatment, along with pro-inflammatory alterations associated with aging, termed “inflammaging” [59]. Furthermore, TNF-α is a crucial pro-inflammatory cytokine that has numerous effects on metabolism and the nervous system. TNF-α increases NF-κB signaling, enhances the generation of reactive oxygen species (ROS), and disrupts mitochondrial ATP synthesis [69], thus leading to cellular energy inefficiency and subsequently CRF. Among cancer survivor populations, elevated levels of TNF-α have been linked to fatigue, depression, and cachexia [14, 61, 70–73]. The findings of this study augment previous evidence by demonstrating that, even among male cancer survivors who have completed primary therapy, greater TNF-α levels are associated with increased fatigue, underscoring the persistent nature of inflammatory dysregulation in CRF.

This study revealed no detectable correlation between IL-8, IL-10, IFN-γ, and CRF. Although some prior studies have implicated both IL-8 [21, 70, 74] and IFN-γ [13, 21] in the pathophysiology of CRF, the lack of correlation in our study likely reflects methodological and biological factors. First, these cytokines sometimes demonstrate transit or context-dependent expression, which may not be reflected in a single baseline measurement. IL-8 is a chemokine implicated in the acute recruitment of neutrophils, which can exhibit rapid fluctuations in response to infection or acute stress [75]. However, this may not accurately represent the persistent, chronic low-grade inflammation typical of CRF. IFN-γ is typically implicated in cellular immunological activation and is generally elevated during acute immune responses [76]; however, it may also be down-regulated or functionally desensitized in long-term cancer survivors, particularly older individuals experiencing immunosenescence. IL-10 is an anti-inflammatory cytokine, and its circulating levels may be influenced by compensatory immune feedback from pro-inflammatory activities [77], which can obscure direct correlations with fatigue severity. The preponderance of prostate cancer survivors in this study, many of whom completed primary cancer treatment, suggests that systemic cytokine activity may have normalized over time, with persistent fatigue primarily resulting from chronic metabolic and mitochondrial dysregulation rather than acute inflammatory cytokines. Differences in treatment, residual hormonal effects, and individual variability in immune recovery may have also contributed to the non-significant findings.

Eleven genes associated with CRF severity were identified in this study, thus providing additional insights into its pathophysiology. The expression of the IRF7 gene was increased in participants experiencing higher fatigue severity. IRF7 primarily regulates type I interferon signaling and antiviral responses [78]. The increased expression may suggest higher interferon-mediated immunologic competence and maladaptive immune remodeling, findings that align with reports of increased interferon-related activity in general chronic fatigue and other inflammation-associated conditions [78]. Consistent with a study exploring gene expression profiling fatigue in female breast cancer patients receiving chemotherapy [46], the expression of the COMMD9 gene decreased as CRF increased in this current study. COMMD9, a member of the copper metabolism Murr1 domain, down-regulates NF-κB activation and modulates hypoxia-inducible factor-1α activity [79]. The decreased expression of COMMD9, associated with decreased NF-κB activation, could result in cytokine production, thereby exacerbating pro-inflammatory signaling in older male cancer survivors.

Similar to the study by Black et al. [22] that included colorectal cancer survivors, we identified an association between the expression of ETS1 and LY6E genes and CRF. The ETS1 gene plays a crucial role in the differentiation of lymphocytes and the regulation of cytokine transcription [80]. Its increased expression indicates a reduction in adaptive immune modulation. LY6E facilitates interferon signaling and the activation of T cells [81]. Its decreased expression further corroborates the hypothesis that immune depletion and compensatory suppression are prevalent in the immunological landscape of cancer survivors with fatigue. OPRL1, a neuroendocrine receptor that regulates stress, nociception, and the HPA axis, has been linked to very high evening fatigue in patients with breast, gastrointestinal, gynecological, or lung cancer undergoing chemotherapy [24], and was upregulated in the present study. Impaired neuroendocrine feedback, which has been reported to be associated with depression and fatigue [24], could occur because of the increased expression of the OPRL1 gene. The expression of ETS1, LY6E, and OPRL1 genes indicates a possible simultaneous activation of pro-inflammatory transcriptional pathways and suppression of counter-regulatory processes in CRF.

CRF was also strongly linked to genes that control mitochondrial and metabolic pathways. The differential expression of these genes demonstrated the impact of mitochondrial malfunction and metabolic abnormalities on CRF. The diminished expression of SNORD89, involved in rRNA modification and cellular stress tolerance, suggests compromised oxidative stress recovery and translational inefficiency in the pathogenesis of CRF [82]. The TFIP11 gene regulates RNA splicing and ribonucleoprotein assembly, which are crucial for maintaining the health of mitochondrial proteins [83]. Therefore, lower levels of TFIP11 expression may indicate altered RNA metabolism, potentially resulting in reduced energy production by the mitochondria. The TGM2 gene has multiple functions. It helps proteins cross-link when they are under oxidative or inflammatory stress, and it also facilitates apoptosis and modifies the extracellular matrix [84]. Higher expression of TGM2 may indicate the body’s response to chronic inflammation, rendering cells less resilient to stress. The reduced expression of the UNG gene, which is crucial for base excision repair [85], was also observed in these fatigued older male cancer survivors, indicating their reduced DNA repair ability and susceptibility to oxidative damage. Combined, these findings suggest that mitochondrial and oxidative stress pathways serve as primary mechanisms connecting inflammation and CRF.

Increased expression of HSPA2 and CXCR4 genes was associated with greater CRF, consistent with the findings reported by Light et al. [29] in their study of prostate cancer patients receiving hormone therapy. HSPA2, a member of the heat shock protein (HSP)-70 family, serves as a molecular chaperone that maintains protein stability during stress conditions [86]. Its increased expression may serve as a compensatory mechanism in response to persistent cellular stress or mitochondrial impairment. CXCR4, a chemokine receptor implicated in leukocyte trafficking and the recruitment of inflammatory cells [87], was also upregulated, thereby reinforcing the concept of ongoing immune activation. Increased CXCR4 gene expression has been observed in chronic inflammatory conditions and may play a role in CRF by sustained signaling within the immune-metabolic axis.

We identified three functional gene clusters associated with CRF based on mRNA intercorrelations among older male cancer survivors. Cluster 1 combined purinergic signaling (P2RY1, P2RX4), metabolic and mitochondrial regulation (ATP5E, DBI), and innate immune stress sensors (TLR4, IRF5–8) with neuroendocrine and circadian regulators (CRY2, OXTR). Together, these genes formed a network linking cellular energy balance and inflammatory initiation, a stress-sensitive, innate gateway signature. Cluster 2 was dominated by cytokine and chemokine signaling (IL1B, IL6, TNF, CXCL2, CXCR4) and rapid-response nuclear factors (NR4A1–3, PER1). It represented a pro-inflammatory transcriptional surge coupled with circadian and immediate-early regulatory control, essentially an acute neuroimmune activation module. Cluster 3 was a diverse cluster that integrated circadian regulators (CLOCK, PER2, SIRT1), mitochondrial and oxidative metabolism genes (NDUFS5, FIS1, BCS1L, SLC25A37), and immune modulators (IRF1–9, NFKB2, NLRC4). These functional results suggested a time-dependent metabolic–immune coordination that mediates adaptive responses to stress and energetic demand.

### Limitations and strengths

Although this study highlighted key findings on the biological correlates of CRF severity among older male cancer survivors, several limitations must be acknowledged. First, while the sample size is relatively larger than those typically used in inflammatory and gene expression studies, future studies with larger samples may help researchers better understand the complex interplay between gene expression profiles and pathways associated with CRF. Power was not calculated a priori, as this was an exploratory analysis. Nevertheless, if the relationships were strong enough to be of interest, they likely would have been statistically significant with confidence intervals excluding the null, as demonstrated by several multiple-testing adjusted significant findings. Our study population was restricted to male cancer survivors who were aged 55 and above, with many of them diagnosed over five years prior, and had completed their initial primary therapy. Consequently, our study’s findings may not apply to younger male cancer survivors or those undergoing cancer treatment. Each participant had at least some level of meaningful fatigue; therefore, those with no fatigue were not assessed. A major cause of CRF in older male cancer survivors (especially prostate cancer) is low (castrate) serum levels of testosterone. However, due to a small sample of patients on ADT or those surgically castrated, our analyses were not stratified by these important variables. Future gene expression profile studies should assess the impact of ADT and testosterone on CRF among this study population. Many gene expression profiles examined in this study did not include protein-level assessment: The observed increase or decrease in mRNA expression does not inherently indicate alterations in the corresponding proteins or receptors, and further research should verify the consistency between mRNA and protein levels. Moreover, the study’s cross-sectional design and single-point analysis limit the ability to infer causal associations in circulating inflammatory markers, gene expression activity, and CRF. Consequently, the correlative findings should be considered exploratory and hypothesis-generating and interpreted with caution. The HERO Trial, however, incorporated serial assessments of the biomarkers, and our forthcoming efforts will examine longitudinal changes in the biomarkers resulting from the Tai Chi Qigong intervention. The extensive inclusion and exclusion criteria for participant enrollment in the trial may result in selection bias. We did not evaluate all potentially important cytokines (e.g., IL-1-ra, IL-1α, and IL-1β). Cytokines not assessed in this study may also influence cytokine-symptom networks. Future studies should assess additional cytokine panels to obtain a more extensive cytokine network. While our report focused on gene expression profiles, further gene analysis, such as DNA methylation and genetic polymorphisms, should be explored. Gene expression was assessed in whole peripheral blood, which may not accurately reflect biological activity at the blood-brain barrier or within specific organs and tissues. The participants were predominantly White and non-Hispanic, with most possessing a college education, which further limits the generalizability of the findings. Male cancer survivors suffering from CRF constitute a heterogeneous group, encompassing individuals affected by frailty, medications, and chronic diseases. Approximately 90% of the participants had prostate cancer; therefore, our findings may not accurately represent CRF in participants with other cancer types. Another limitation is that cytokines seldom function independently in vivo; each cytokine is closely associated with several upstream and downstream proteins and factors within the inflammatory cascade. Consequently, novel computational bioinformatics techniques, such as network analysis or latent class analysis, may be required to achieve a more comprehensive understanding of cytokine effects on CRF.

Despite these inherent limitations, this study has several noteworthy strengths. This biological correlation study was conducted among male cancer survivors, a group that is less extensively studied compared to their female counterparts. The sample size is larger than typically observed in inflammatory marker and gene expression research, including among cancer survivors. Novel functional gene expression clusters associated with CRF were identified in this study population. These clusters may serve as molecular targets to further our understanding of the pathophysiology of CRF and the biological and/or medical basis of interventions that might ultimately reduce clinical CRF levels.

## Conclusion

Worse CRF was associated with inflammatory profiles and coordinated shifts in expression of genes involved in immune activation, neuro-immune signaling, mitochondrial dysfunction, and nucleic acid processing among older male cancer survivors. Taken together, these inflammatory and gene expression profile patterns are consistent with systemic inflammatory activation, perturbations in circadian regulation, disruptions in cellular energy metabolism and RNA/DNA maintenance, offering a coherent biological context for CRF in this population. The identification of functional gene clusters establishes a biological foundation for subsequent CRF mechanistic studies. mRNA genes unique to functional clusters may serve as diagnostic markers for CRF susceptibility or as endpoints for possible molecular targets in interventions designed to reduce CRF and enhance cancer survivorship.

## Supplementary information

Supplementary information

## Data Availability

The datasets used and/or analyzed during the current study are de-identified and available from the corresponding author on reasonable request.
