# Supplementary material for: Biological correlates of cancer-related fatigue in older male cancer survivors
Source: Transl Psychiatry. 2026 Jul 17;16:551. doi: 10.1038/s41398-026-04274-1 (PMC13658807; doi:10.1038/s41398-026-04274-1)
Supplement: Supplementary file 1 [file 41398_2026_4274_MOESM1_ESM.docx]

**Biological correlates of cancer-related fatigue in older male cancer survivors**

**SUPPLEMENTARY INFORMATION**

**S Table 1.** Genes examined by their pathway

**S Table 2.** Correlation between circulating inflammatory markers and severity of fatigue.

**S Figure 1.** Functional gene clusters based on mRNA intercorrelations with three clusters identified.

**S Table 1. Genes examined by their pathway**

| **Pathways** | **Genes** |
| --- | --- |
| Adrenergic, monoamines, peptides | ADRB2 (↓), COMT (↓), NR3C1 (↑), NR4A2 (↑), NR4A3 (↑), OPRL1 (↑), OXT (↓), OXTR (↓), VIPR2 (↓), GNAI2 (↑), UTS2 (↑) |
| Immune response and inflammation | AIG1 (↑), ANXA2 (↑), ARG1 (↑), CCL20 (↑), CCR7 (↑), CD14 (↑), CD27 (↑), CD28 (↑), CD70 (↑), CXCL2 (↑), CXCR4 (↑), CXCR5 (↑), CYBB (↑), EGR1 (↑), GADD45A, (↑), HLA-H (↑), HMGB2 (↑), HMOX1 (↑), HOXA9 (↑), LY6E (↓), COMMD9 (↓), IFI27 (↑), IFI6 (↑), IL-10 (↓), IL-1A (↑), IL-1B (↑), IL-1R1 (↑), IL-1R2 (↑), IL-4 (↓), IL-6 (↑↓), IRAK3 (↑), IRF1 (↑), IRF2 (↑), IRF3 (↑), IRF4 (↑), IRF5 (↑), IRF6 (↑), IRF7 (↑), IRF8 (↑), IRF9 (↑), ISG15 (↑), ITGAX (↑), KLRC1 (↑), LILRA6 (↑), LTA (↑), LTB (↑), LYN (↑), MAPK14 (↑), MEFV (↑), MS4A1 (↑), NCF1 (↑), NFKB2 (↑), NLRC4 (↑), OSM (↑), PRDX6 (↑), S100Z (↑), SESN1 (↑), SESN2 (↑), SESN3 (↑), SH3RF1 (↑), SIRT1 (↑), SNCA (↑), ST5 (↑), TGFB1 (↑), TGM2 (↑), TIMP2 (↑), TLR4 (↑), TNF (↑), UBD (↑), UBE2V1 (↑), VEGF (↑). |
| Mitochondria and lipid/energy metabolism | AIFM2 (↑), ATP5E (↓), BCS1L (↑), CYBB (↓), DBI (↓), ECH1 (↑), FIS1 (↓), HBA1 (↑), HBA2 (↑), HBB (↑), HSPA2 (↑), HBG2 (↑), IMMP2L (↓), NDUFS5 (↑), PRDX6 (↓), RXRA (↓), SLC25A23 (↑), SLC25A37 (↑), SOD2 (↓), TXNRD2 (↓), TGM5 (↓), TRMT2A (↑), TFIP11 (↓), SESN2 (↓) |
| Sensory ion channels (ATP- and metabolite sensing) | ASIC3 (↓), P2RX4 (↓), P2RX7 (↓), P2RY1 (↑), CA1 (↑). |
| Transcription and growth factors | ARNTL2 (↑), ATF4 (↑), CAPRIN2 (↑), CCNJ (↑), CLOCK (↓), CRY2 (↑), DUSP18 (↑), EIF1 (↑), HIST1H4E (↑), IRF9 (↑), JUND (↑), KMT2E (↑), NCOA1 (↑), ETS1 (↑), NR3C1 (↑), PER1 (↓), PER2 (↓), SNORD89 (↓), SETD1A (↑), SIRT1 (↑), TFPI (↑), TAF1C (↑), TCEAL1 (↑), TGFB1 (↑), TJP2 (↑), TFIP11 (↓) , VIPR2 (↑), UNG (↓) , YIF1B (↑), ZNF331 (↑), ZNF607 (↑), ZNF788 (↑). |

(↑) Increased expression is hypothesized to be associated with CRF

(↓) Decreased expression is hypothesized to be associated with CRF

**S Table 2. Correlation between circulating inflammatory markers and severity of fatigue**

| **Biomarkers^a^** | **Bivariate** | | **Multivariable^b^** | |
| --- | --- | --- | --- | --- |
|  | **r (95% CI)^c^** | **p-value** | **β (95% CI)** | **p-value** |
| CRP | -0.24 (-0.41 - -0.06) | 0.011 | -0.04 (-0.08 - -0.02) | 0.003 |
| IFN- γ | 0.08 (-0.12 – 0.26) | 0.435 | 0.01 (-0.01 – 0.03) | 0.293 |
| IL-10 | -0.05 (-0.24 – 0.14) | 0.597 | 0.01 (-0.02 – 0.02) | 0.772 |
| IL-6 | -0.29 (-0.46 - -0.11) | 0.002 | -0.03 (-0.05 - -0.01) | <0.001 |
| IL-8 | 0.05 (-0.14 – 0.23) | 0.635 | -0.01 (-0.02 – 0.01) | 0.538 |
| TNF- α | -0.25 (-0.42 - -0.06) | 0.010 | -0.01 (-0.02 - -0.001) | 0.028 |

^a^circulating inflammatory markers have been log-transformed

^b^ adjusted for age, race, ethnicity, marital status, education, income, health insurance, BMI, cancer type, years since diagnosis, stage of disease, and use of hormone therapy.

^c^ 95% CI calculated with z Fisher’s transformation.

**S Figure 1. Functional gene clusters based on mRNA intercorrelations with three clusters identified.** abs(r) = strength of association and as.factor(cluster) = functional clusters.
